# Subjective emotional arousal: an explorative study on the role of gender, age, intensity, emotion regulation difficulties, depression and anxiety symptoms, and meta-emotion

**DOI:** 10.1007/s00426-019-01197-z

**Published:** 2019-05-16

**Authors:** Matthias Deckert, Michaela Schmoeger, Eduard Auff, Ulrike Willinger

**Affiliations:** Department of Neurology, Medical University of Vienna, Waehringer Guertel 18-20, 1090 Vienna, Austria

## Abstract

Subjective emotional arousal in typically developing adults was investigated in an explorative study. 177 participants (20–70 years) rated facial expressions and words for self-experienced arousal and perceived intensity, and completed the Difficulties in Emotion Regulation scale and the Hospital Anxiety and Depression scale (HADS-D). Exclusion criteria were psychiatric or neurological diseases, or clinically relevant scores in the HADS-D. Arousal regarding faces and words was significantly predicted by emotional clarity. Separate analyses showed following significant results: arousal regarding faces and arousal regarding words constantly predicted each other; negative faces were predicted by age and intensity; neutral faces by gender and impulse control; positive faces by gender and intensity; negative words by emotional clarity; and neutral words by gender. Males showed higher arousal scores than females regarding neutral faces and neutral words; for the other arousal scores, no explicit group differences were shown. Cluster analysis yielded three distinguished emotional characteristics groups: “emotional difficulties disposition group” (mainly females; highest emotion regulation difficulties, depression and anxiety scores; by trend highest arousal), “low emotional awareness group” (exclusively males; lowest awareness regarding currently experienced emotions; by trend intermediate arousal), and a “low emotional difficulties group” (exclusively females; lowest values throughout). No age effect was shown. Results suggest that arousal elicited by facial expressions and words are specialized parts of a greater emotional processing system and that typically developing adults show some kind of stable, modality-unspecific dispositional baseline of emotional arousal. Emotional awareness and clarity, and impulse control probably are trait aspects of emotion regulation that influence emotional arousal in typically developing adults and can be regarded as aspects of meta-emotion. Different emotional personality styles were shown between as well as within gender groups.

## Introduction

### The role of emotional arousal in emotion processing

Emotional responses can be defined as reactions to evocative stimuli, in terms of identifying the emotional significance of a stimulus or situation, producing an affective state, and regulating the affective state (Phillips, Drevets, Rauch, & Lane, 2003). These responses involve aspects like, for example, physiological arousal, goal-directed behavior, expressive behavior, cognitive appraisal, or subjective experience (Blascovich & Tomaka, 1996; Borod, 1993; Borod et al., 2000; Murray et al., 2015; Phillips et al., 2003; Plutchik, 1984). In emotion processing, arousal plays a central role as it was stated that regarding the perception of emotional stimuli only the dimensions arousal and valence (pleasure) can be seen as relatively culture-free classification dimensions (Russell, 1994). Furthermore, “attention to and effortful regulation of arousal” is seen as a central aspect of emotion regulation (Phillips et al., 2003, pp. 509). To date, arousal is defined as the degree of excitement or motivational activation (Bradley, Codispoti, Sabatinelli, & Lang, 2001a; Lang, Bradley, & Cuthbert, 1999) a person experiences as a reaction to emotional stimuli. Valstar (2015) defines arousal as a global feeling of dynamism or lethargy that involves mental activity and physical preparedness to act.

In everyday life, individuals encounter a great number of emotive stimuli like emotional faces and emotional words, especially when having face-to-face conversations. As these stimuli play a very important role in everyday social life (personal encounters, messages, social media (images of faces, profile pictures, statements, etc.), and so on) and as it was shown that emotional facial expressions as well as emotional words enhance arousal compared to neutral ones (Regenbogen et al., 2012; Voe, Jacobs, & Conrad, 2006), the following article will mainly focus on these emotive stimuli.

To sum up, arousal is a fundamental aspect of everyday emotion perception and emotion regulation, whereas emotional faces and emotional words are on the one hand ideal representatives of natural everyday emotional stimuli and on the other hand excellent tools for the investigation of emotional arousal.

### Emotional arousal: influential variables

#### Age

With respect to emotional arousal across the life span, age differences regarding trait emotional processing can be seen. In this context, it was shown that older adults compared to younger adults in general show a decreased negative trait arousal and an increased low arousal trait positive affect (Kessler & Staudinger, 2009) while generally tending to pay more attention to and remember more positive information (Isaacowitz et al., 2006). Nevertheless, when actually confronted with emotional pictorial stimuli, age differences also occur regarding state emotional processing, as older adults rate negative pictures as more arousing and positive pictures as less arousing (Grühn & Scheibe, 2008).

Possible reasons for such differences in emotional arousal presumably lie within developmental changes in trait and state aspects of emotion regulation (e.g., Dolcos, Katsumi, & Dixon, 2014; Kessler & Staudinger, 2009; Mather & Johnson, 2000). Age differences in trait aspects comprise, for example, subjective ratings of your own emotion regulation behavior, emotion regulation abilities and tactics, as well as neurobiological disposition. In this context, older adults show advantages in perceived affect regulation and report being better at controlling their emotions (Gross et al., 1997; Kessler & Staudinger, 2009), show an increased emotion regulatory capacity and emotional problem solving ability (Blanchard-Fields, 2007; Urry & Gross, 2010), and potentially can be seen as chronic emotion regulators who show chronically activated neuronal emotion regulation networks (Dolcos, Katsumi, & Dixon, 2014; Mather & Johnson, 2000).

Nevertheless, it was shown that arousal levels moderate age differences in emotion processing (Kappes & Bermeitinger, 2016) and that the effectiveness of specific emotion regulation strategies is significantly influenced by the arousal-inducing potential of a stimulus or condition (e.g., Fitzpatrick & Kuo, 2016; Shafir et al., 2015). In this context, it has to be noted that older adults likely constantly pursue goal-directed processes to achieve long-term emotional well-being goals (“Socioemotional Selectivity Theory” by Carstensen, 2006; but also e.g., Kryla-Lighthall & Mather, 2009, Livingstone & Isaacowitz, 2015), a process that is seemingly characterized by, inter alia, stable relations between past emotional behavior and current emotion processing (see, e.g., Kappes & Bermeitinger, 2016). In this way, older adults generally show more effective emotion regulation performances (Charles, 2010) as they, for example, spend less time with negative arousing emotional stimuli than younger adults (Livingstone & Isaacowitz, 2015). This age-related superiority seemingly vanishes when older adults experience sustained high-level emotional arousal and have more difficulties returning to an emotional “baseline” (see, e.g., “Strength and Vulnerability Integration Model”—SAVI, Charles, 2010).

These aspects presumably add to age differences in the perception of everyday emotional stimuli. Regarding the perception of facial expressions, for example, Svard, Fischer, and Lundqvist (2014) showed that older adults compared to younger adults perceive less arousal regarding a single averaged, morphed, angry male face as well as a female face, respectively. In contrast, Deckert (2014) showed no age differences regarding natural angry faces as well as neutral, happy, sad, and fearful faces while using a different rating scale than Svard et al. (2014). It has to be noted that in the study of Svard et al. (2014), the rating took place after processing emotional faces in three different preceding tasks, whereas in Deckert (2014) participants either rated faces in the first place or after rating emotional words. Regarding emotional words, some studies indicate an age-related relation between valence and arousal (e.g., Bjalkebring & Johansson, 2015; Yang & Hasher, 2011). For instance, it was shown that older adults rate their happiness higher when being previously presented with low arousing framing words and rated happiness lower when being presented with high arousing framing words, whereas this effect was not shown in younger adults (Bjalkebring & Johansson, 2015). Furthermore, Yang and Hasher (2011) showed that in a speed word fragment completion task, older adults show an accessibility bias towards neutral words, whereas younger adults favored positive words. In contrast, Deckert (2014) showed no age-related arousal effects, as no differences were shown between younger, middle, and older adults regarding the arousal rating of negative and neutral words.

To sum up, inconsistent results regarding age-related differences in emotional arousal as a reaction to emotional faces and words can be seen. Besides age-related differences in state and trait aspects of emotional arousal perception and regulation, different study designs and methodology used in studies can be seen as potential explanations for these results.

#### Gender

With respect to gender, literature indicates that females are more reactive to unpleasant events and show a broad disposition to respond with greater arousal to emotional stimuli, especially unpleasant ones (Bradley, Codispoti, Sabatinelli, & Lang, 2001b; Gomez, von Gunten, & Danuser, 2013; Deng, Chang, & Yang, 2016). Studies investigating gender differences in arousal regarding emotional faces are scarce. In this context, Thayer and Johnsen (2000) hypothesize that females compared to males generally rely more on subtle differences in expressed arousal when processing facial expressions, while Deckert (2014) shows no gender differences in subjective arousal regarding facial expressions. Regarding emotional words, Soares, Comesana, Pinheiro, Simoes, and Frade (2012) showed gender differences in arousal in a sample of students who rated negative, neutral, and positive Portuguese words as females showed higher arousal scores. Deckert (2014), however, showed no gender differences in similarly aged as well as elder participants with respect to the rating of negative and neutral words whereby the sample showed more heterogeneity regarding education.

Again, besides differences in study design, methodology, and sample, gender-specific differences in regulating arousing emotional stimuli should be taken into account. With respect to emotion regulation and experienced arousal, for example, no gender differences were found on the behavioral level (McRae, Ochsner, Mauss, Gabrieli, & Gross, 2008). Despite the absence of overt behavioral gender differences, physiological and neuronal differences between males and females were shown (Kim, 2005; McRae et al., 2008). In this context, Kim (2005) showed that when cognitively decreasing negative emotions, males showed a trend of increased physiological arousal, whereas females did not. Furthermore, McRae et al. (2008) showed that when cognitively down-regulating emotions, females showed a greater engagement of brain regions associated with reappraisal, emotional responding, and reward processing.

To sum up, a paucity of research addressing gender effects regarding emotion regulation and subjective arousal leaves the necessity for new data.

#### Intensity

Within individuals, genuine (non-inhibited) facial expressions represent emotional states that are defined by a certain degree of arousal (and valence), whereas their appearances depend on the intensity with which the individual’s facial muscles are activated (see e.g., Cohn, Ambadar, & Ekman, 2007; Ekman, 2003, Goeleven et al., 2008). Whereas this relation between arousal and intensity within an individual is reasonable because of a direct neurobiological link (see e.g., Phillips et al., 2003), the relation between another person’s facial expression’s intensity and an observer’s arousal could be diminished due to influencing factors like, for example, emotion regulation efficiency and context. Nevertheless, despite a dearth of studies dealing with this topic, a few studies indicate a moderate to strong relation between the subjective emotional arousal an individual experiences when observing an emotional face and the level of intensity he or she attributes to this emotional face (Deckert, 2014; Goeleven et al., 2008). Furthermore, in their defense cascade model, Bradley et al. (2001a, b) postulate that defense motivation in terms of unpleasant arousal increases with the intensity of a perceived threat.

To sum up, there is a paucity of studies investigating the relationship between experienced subjective emotional arousal as a reaction to emotional faces and the intensity that is attributed to these faces.

#### Difficulties in emotion regulation

Emotion regulation can be defined as the context-dependent (Gross & Thompson, 2007) ability to modulate the magnitude or duration of an emotional response (Gross, Sheppes, & Urry, 2011) in terms of “attention to and effortful regulation of arousal” (Phillips et al., 2003, pp. 509). It requires the activation of an intrinsically or extrinsically activated goal (Gross & Thompson, 2007) and uses a continuum of (mental) operations (see e.g., Gyurak, Gross, & Etkin, 2011); for a review see Gross (2013).

Viewing emotion regulation as a complex, heterogeneous operation, Gratz and Roemer (2004) suggest six dimensions in which difficulties can occur: impulse control, emotional clarity, goal-directed behavior, emotional awareness, acceptance of emotional responses, and access to emotion regulation strategies. With respect to impulse control, in terms of successful inhibition of inappropriate or impulsive behaviors (Gratz & Roemer, 2004), a relation with arousal can be drawn, as impulsiveness influences the emotional modulation of response inhibition (Benvenuti et al., 2015) and can be seen in diseases like, e.g., posttraumatic stress disorder (PTSD—Short et al., 2016). Emotional clarity, defined as the extent to which an individual is clear about the emotions he or she is currently experiencing (Gratz & Roemer, 2004), was shown to predict arousal discrimination ability (Nielsen, 2004). Problems with emotional clarity can be found in diseases that involve abnormal physiological arousal like, for example, depression and social anxiety (see e.g., APA, 2013; but also Short et al., 2016; Vine & Aldao, 2014). In this context, alexithymia and its different types are associated with malfunctioning arousal processing like hypo- or hyper-reactivity to emotional stimuli and problems in interpreting these physiological reactions (e.g., Larsen et al., 2003). In the previously mentioned definition (e.g., Borod, 1993), goal-directed behavior and arousal were stated to be important aspects of emotional processing. Goal-directed behavior, defined as the ability to concentrate as well as to accomplish tasks when experiencing negative emotions (Gratz & Roemer, 2004), was thought to be part of a dynamic system of emotion regulation (Kuhl, 1983). Furthermore, a recent review indicates that in emotion processing, goal-directed processes in terms of appraisal are superior to stimulus-driven processing and regulation (Moors, Boddez, & De Houwer, 2017). In this way, goal-directed processes regulate emotional behavior by refining and correcting action tendencies in emotion processing (Moors, Boddez, & De Houwer, 2017). Difficulties with respect to the non-acceptance of emotional responses, defined as the “tendency to have negative secondary emotional responses to one’s negative emotions or non-accepting reactions to one’s distress” (Gratz & Roemer, 2004, pp. 47), were shown to predict symptoms of diseases that involve abnormal physiological arousal like, for example, depression (e.g., APA, 2013; Lilly & London, 2015). Self-referential emotional awareness, defined as the “tendency to attend to and acknowledge emotions” (Gratz & Roemer, 2004, pp. 47), was shown to attenuate emotional arousal (Herwig, Kaffenberger, Jancke, & Bruhl, 2010). Difficulties regarding access to emotion regulation strategies which is defined as the subjective belief that emotion regulation strategies cannot be initiated effectively when you get upset (Gratz & Roemer, 2004), can also be seen in diseases that involve abnormal physiological arousal like, for example, bipolar disorder (APA, 2013; Salsman & Linehan, 2012; Van Rheenen, Murray, & Rossel, 2015).

To date, there is a dearth of studies (e.g., Benvenuti et al., 2015; Herwig et al., 2010; Nielsen, 2004) focusing on the link between emotional arousal and specific emotion regulation difficulties in healthy individuals or subclinical populations, respectively. Furthermore, whereas this link was previously studied with respect to single emotion regulation difficulty dimensions, to the knowledge of the authors, no study so far addressed this link regarding all six dimensions proposed by Gratz and Roemer (2004).

To sum up, only loose connections between emotion regulation difficulties and subjective arousal can be found, whereas the majority of data comes from patient samples.

### Aim of the study

Arousal is a fundamental aspect of everyday emotion perception and emotion regulation. So far, only a few studies have addressed subjective emotional arousal in typically developing individuals, therefore this study aimed to offer new, explorative data with respect to this topic. To offer results with high everyday relevance (e.g., social encounters, social media), processing of emotional faces and words was investigated as those are ideal representatives of natural everyday emotion stimuli. Furthermore, these stimuli were shown to be excellent tools for the investigation of emotional arousal, as both emotional faces and emotional words show significantly higher arousal than neutral ones. These stimuli were further chosen so as to offer new data with respect to specific research questions which yielded inconsistent results so far. In everyday life, these stimuli very often appear together like, for example, in face-to-face conversations. Therefore, another aim was to investigate whether results suggest that subjective emotional arousal processing transcends the processing of single stimulus types, in terms of a general arousal “behavior”. In this context, similar to Blascovich and Tomaka (1996) who suggested that individuals show dispositional physiological response levels and dispositional somatosensory sensitivity, another aim of the study was to investigate whether arousal ratings regarding fundamental stimulus types (i.e., faces and words) show associations, similar patterns, and/or shared predictors that could be interpreted as some sort of dispositional baseline of subjective emotional arousal. Due to the dearth of studies focusing on this topic, the current study aimed to present new data regarding such a possible emotional personality trait in typically developing individuals.

So far, there is a dearth of studies that investigate possible meaningful predictors of subjective emotional arousal in typically developing adults like, for example, age, gender, intensity, emotion regulation difficulties, or symptoms of diseases associated with emotion regulation problems like depression or anxiety. To date, inconsistent results regarding age- and gender-related differences in emotional arousal as a reaction to emotional faces and words were shown. Furthermore, so far, arousal has been extensively connected to emotion regulation and regulation techniques, but only loose connections with emotion regulation difficulties can be found, whereas the majority of studies focused on patient samples. In addition, whereas arousal and emotion regulation difficulty problems can be seen in depression and anxiety, relatively few is known with respect to the role of subclinical degrees regarding subjective emotional arousal. Therefore, based on literature and resulting theoretical considerations, another aim of this study was to investigate whether subclinical degrees are predictive of subjective emotional arousal in typically developing adults. For that purpose, participants with clinically relevant anxiety and depression symptoms were excluded from the study (see the “Participants” section). Although this possibly leaves low variability in these scores, these aspects were investigated to give a more holistic view on subjective emotional arousal. It has to be noted that these scores were added to the analyses in this study in the course of a re-analysis of the dataset that was inspired by the reviews of the manuscript.^1^ Furthermore, existing studies on emotional arousal mostly deal with single factors and show unclear data, whereby differences in study design and methodology lead to a decreased comparability of results. Therefore, another aim of the study was to include the previously mentioned predictors within a single study design as well as within different combined analyses. This was done so as to enable statements regarding the relative impact of each factor regarding emotional arousal and to investigate possible meaningful combinations of these predictors in typically developing individuals across adulthood. Besides calculations using an emotional arousal total score, the predictive value of the previously mentioned variables was tested for faces and words separately which were either positively arousing, negatively arousing, or neutral. This was done as it was previously shown that the influence of predictors, such as age, on arousal depends on the valence of the stimuli and so as to compare the processing of emotional stimuli with that of neutral stimuli in typically developing adults as at least age-related effects on neutral stimulus processing were previously shown.

The final aim of this study was to look whether distinguishable subjective emotional arousal profiles can be found within the current sample. In the light of previous approaches on identifying groups differing in emotional processing characteristics (e.g., Labouvie-Vief, 1998; Labouvie-Vief & Marquez, 2004), the current study aimed to explore possible emotional personality styles, based on emotional arousal and the previously mentioned variables as so far, only a few studies addressed these aspects in a combined way in typically developing individuals across the age span. Given this explorative approach as well as the inconsistent results in literature, emotional arousal profiles relying strongly on either age or gender or a combination of both are conceivable. Age effects could lead to decreasing arousal levels, whereas females could show a higher disposition to react with greater arousal to emotional stimuli. As this is the first study to investigate the effect of different aspects of emotion regulation difficulties as well as depression and anxiety symptoms in typically developing adults, either general higher emotional difficulties (emotion regulation difficulties, depression and anxiety symptoms) or unique combinations of these aspects are possible across groups.

### Research questions

Given the inconsistent or missing results in literature, all research questions are unspecific and rather of explorative nature.

*Research question 1* Is there a significant prediction of subjective emotional arousal as a reaction to the perception of facial expressions and words by age, gender, emotion regulation difficulties, depression symptoms, and anxiety symptoms?

*Research question 2* Is there a significant prediction of subjective emotional arousal as a reaction to the perception of negative facial expressions by age, gender, subjective emotional arousal elicited by words, intensity ratings of facial expressions, emotion regulation difficulties, depression symptoms, and anxiety symptoms?

*Research question 3* Is there a significant prediction of subjective emotional arousal as a reaction to the perception of neutral facial expressions by age, gender, subjective emotional arousal elicited by words, intensity ratings of facial expressions, emotion regulation difficulties, depression symptoms, and anxiety symptoms?

*Research question 4* Is there a significant prediction of subjective emotional arousal as a reaction to the perception of positive facial expressions by age, gender, subjective emotional arousal elicited by words, intensity ratings of facial expressions, emotion regulation difficulties, depression symptoms, and anxiety symptoms?

*Research question 5* Is there a significant prediction of subjective emotional arousal as a reaction to the perception of negative words by age, gender, subjective emotional arousal elicited by faces, emotion regulation difficulties, depression symptoms, and anxiety symptoms?

*Research question 6* Is there a significant prediction of subjective emotional arousal as a reaction to the perception of neutral words by age, gender, subjective emotional arousal elicited by faces, emotion regulation difficulties, depression symptoms, and anxiety symptoms?

*Research question 7* Can distinctive profiles of emotional arousal be found, based on arousal as a reaction to facial expressions, arousal as a reaction to words, age, gender, intensity ratings of facial expressions, emotion regulation difficulties, depression symptoms, and anxiety symptoms?

## Method

### Participants

The sample consisted of 177 typically developing participants (females: 55%, males: 45%) aged between 20 and 70 years (*M* = 44.3, SD = 16.8 years). The sample size was calculated with G*Power (linear multiple regression, effect size *f*^2^ = 0.15, *α* = 0.05, power 1 − *β* = 0.95; Faul et al. 2007). The single age groups can be described as follows: 40 (23%) participants in emerging adulthood aged between 20 and 25 years (57% female), 32 (18%) participants in early adulthood aged between 26 and 40 years (47% female), 48 (27%) participants in the first half of middle adulthood aged between 41 and 50 years (54% female), 25 (14%) participants in the second half of middle adulthood aged between 54 and 64 (76% female), and 32 (18%) participants in late adulthood aged between 65 and 70 years (44% female), age groups in accordance with Berk (2014), Feldman (2017), and Steinberg (2016). 168 participants (95%) were native German speakers, whereas 9 (5%) participants were very fluent in German (native languages: Croatian, Icelandic, Russian, Hungarian, Slovenian, Persian, and Polish). All participants spent the majority of their adulthood in Austria. 21 (12%) participants completed compulsory school, 6 (3%) completed an apprenticeship, 33 (18%) graduated from a professional school, 2 (1%) completed a lower school type without a high school certificate, 73 (41%) had a high school certificate, 10 (7%) graduated from an academy, 6 (3%) had a college degree, and 26 (15%) had an university degree. 47 (27%) participants were current university students, whereas 130 (73%) did not study. 110 (62%) participants were currently employed with a mean weekly working time of 33 h (SD = 14.2), whereas 67 (38%) were unemployed or already retired. 91 (51%) participants were Catholics, 70 (40%) had no confession of faith, 13 (7%) were Evangelic, whereas 3 (2%) had other religious confessions. These characteristics indicate a representative sample of the country studied in (Austria). In the sociodemographic form, participants were asked whether they currently suffer from a neurological or psychiatric disease or did so in the past. No participant reported a history of or a current disease of that kind. Using the German version of the Hospital Anxiety and Depression Scale (HADS-D—Herrmann-Lingen, Buss, & Snaith, 2011), participants were initially screened for anxiety and depression symptoms which yielded a mean depression score of 2.5 (SD = 2.3) and a mean anxiety score of 4.4 (SD = 2.4). Four participants showed clinically relevant anxiety scores (a score of 11 or greater) and were therefore excluded from further analyses (cutoff scores: 0–7 no clinical signs, 8–10 borderline, 11–14 severe, 15–21 very severe). The study protocol was approved by the Institutional Review Board of the Medical University of Vienna and meets the ethical principles of the Declaration of Helsinki as well as the APA ethical standards for human research. Prior to participation, a written informed consent form was signed by every participant. Participants were recruited by the authors of the study and obtained no incentives.

### Procedures and materials

#### Emotional faces: arousal and intensity rating task

In a computerized task, participants had to rate faces displaying the emotions happiness, fear, anger, and sadness as well as neutral expressions with a straight gaze, taken from the Karolinska Directed Emotional Faces Database (KDEF—Lundqvist, Flykt, & Oehman, 1998). Each emotion as well as the neutral expressions was displayed by female and male photo models yielding a sum of 75 pictures. These 75 pictures of facial expressions were chosen for the current study, because they were previously shown to be valid for the population of Austria Deckert (2014); for cultural differences in general as well as emotional face processing see, for example, Elfenbein and Ambady (2002), or Hills and Lewis (2011). In the current study, participants had to rate for self-experienced arousal as well as the intensity of the displayed expression. First, participants saw the faces in the upper half of the screen, whereas the question “How great is your inner arousal viewing this emotion?” was presented below. Participants rated their personal inner arousal using a combined nine-point Self-Assessment Manikin (SAM—Bradley & Lang 1994) visual scale and nine-point Likert scale ranging between 1 (“low arousal”) and 9 (“high arousal”) with 5 (“middle”) as midpoint. For each face, the SAM and the Likert scale were placed one above the other and participants had free choice in which one to click on. In this way, the rating task yielded separate arousal scores for each type of expression (AROUSAL FACES HAPPINESS, FEAR, ANGER, SADNESS, NEUTRAL). These scores were summed up to an arousal score for negative faces (AROUSAL FACES NEGATIVE, consisting of fear, anger, and sadness), an arousal score for neutral faces (AROUSAL FACES NEUTRAL), and an arousal score for positive faces (AROUSAL FACES POSITIVE, consisting of happiness). These scores again were summed up to an arousal total score (AROUSAL FACES TOTAL). Second, participants saw the faces in the upper half of the screen whereas the question “How intense is this emotion displayed?” was presented below. Participants rated the intensity of the displayed emotion using a nine-point Likert scale ranging between 0 (“no intensity) and 8 (“high intensity”) with 4 (“middle”) as midpoint. The scores were summed up to an intensity score for negative faces (INTENSITY FACES NEGATIVE, consisting of fear, anger, and sadness), an intensity score for neutral faces (INTENSITY FACES NEUTRAL), and an intensity score for positive faces (INTENSITY FACES POSITIVE, consisting of happiness). Scores for the negative and positive faces plus the neutral expressions were added up yielding an intensity total score (INTENSITY FACES TOTAL). In a standardized instruction, each rating scale was explained and participants were invited to ask whenever something was unclear. The stimuli were presented in a fixed order and without a time limit, whereas each facial expression was first rated for personal inner arousal and afterwards rated for its intensity before a new facial expression appeared.

#### Emotional words: arousal rating task

In a computerized task, participants had to rate 100 negative as well as 100 neutral words, taken from the Berlin Affective Word List Reloaded (BAWL-R—Voe et al., 2006). These words were chosen for the current study, because they were previously shown to be valid for the population of Austria Deckert (2014), especially with respect to their emotional connotation. For cultural differences in word processing see, for example, Redondo, Fraga, Padron, and Comesna (2007), Schmidt-Atzert and Park (1999), Soares et al. (2012), or Wierzbicka (2010). Deckert (2014) chose the words in the following way: first, the selection was limited to words within a range of five to seven letters. In the second step, words were chosen based on the valence ratings provided by Voe et al. (7-point scale ranging between − 3 (“very negative) and 3 (“very positive) with 0 (“neutral”) as midpoint). The negative words were within the most negative 25% of the scale (ratings between − 3.0 and − 1.6), as all words with ratings between − 3.0 and − 2.0 were automatically chosen (56 words) and from the range between − 2.0 and − 1.6 44 words were chosen randomly. The neutral words were randomly chosen within a range of − 0.1 and + 0.1. Participants saw the words in the upper half of the screen, whereas the question “How great is your inner arousal reading this word?” was presented below. Participants rated their personal inner arousal using a combined nine-point Self-Assessment Manikin (SAM—Bradley & Lang, 1994) visual scale and nine-point Likert scale ranging between 1 (“low arousal”) and 9 (“high arousal”) with 5 (“middle”) as midpoint. For each word, the SAM and the Likert scale were placed one above the other and participants had free choice in which one to click on. The rating task yielded separate arousal scores for negative (AROUSAL WORDS NEGATIVE) and neutral words (AROUSAL WORDS NEUTRAL) which were summed up to an arousal total score (AROUSAL WORDS TOTAL). In a standardized instruction, each rating scale was explained and participants were invited to ask whenever something was unclear. The stimuli were presented in a fixed order and without a time limit.

#### Difficulties in emotion regulation

Difficulties with emotion regulation were measured using a German version of the difficulties in emotion regulation scale (DERS—Gole, Koechel, Schaefer, & Schienle, 2012; Gratz & Roemer, 2004). Comprising 36 items, the DERS yields six scales: impulse control difficulties (IMPULSE, Cronbach’s Alpha = 0.86), lack of emotional clarity (CLARITY, Cronbach’s Alpha = 0.84), difficulties engaging in goal-directed behavior (GOALS, Cronbach’s Alpha = 0.89), lack of emotional awareness (AWARENESS, Cronbach’s Alpha = 0.80), non-acceptance of emotional responses (NON-ACCEPTANCE, Cronbach’s Alpha = 0.85), and limited access to emotion regulation strategies (STRATEGIES, Cronbach’s Alpha = 0.88). Following example items should illustrate the different scales of the DERS: “When I’m upset, I lose control over my behaviors” (impulse), “I am confused about how I feel“(CLARITY), “When I’m upset, I have difficulty focusing on other things” (GOALS), “I am attentive to my feelings* “(AWARENESS, *reversed item), “When I’m upset, I feel guilty for feeling that way” (NON-ACCEPTANCE), “When I’m upset, I believe that there is nothing I can do to make myself feel better” (STRATEGIES). Higher values in each scale mean greater difficulties in emotion regulation. The DERS total score was not included in the analyses, due to studies which suggested that the use of the total score may not be appropriate for the description of emotion regulation difficulties (Lee et al. 2016).

#### HADS-D

The German version of the Hospital Anxiety and Depression Scale (HADS-D—Herrmann-Lingen, Buss, & Snaith, 2011) is a short questionnaire that assesses anxiety and depression symptoms in the past seven days. Participants are presented with seven statements regarding anxiety symptoms and seven statements regarding depression symptoms in an alternating order with each statement accompanied by four different answer options (0, 1, 2, or 3 points for each answer). The statements are either formulated highlighting the presence of a symptom [“I get a sort of frightened feeling as if something awful is about to happen”—answers: “Not at all” (0 points), “A little, but it doesn’t worry me” (1 point), “Yes, but not too badly” (2 points), “Very definitely and quite badly” (3 points)] or highlighting the absence of a symptom [“I can laugh and see the funny side of things”, answers: “As much as I always could” (0 points), “Not quite so much” (1 point), “Definitely not so much now” (2 points), “Not at all” (3 points)]. The questionnaire yields an anxiety as well as a depression score, whereas the more points a participant obtains in each score, the more pronounced the symptoms. The cutoffs for each score indicate symptoms that are sub-clinical (0–7), borderline (8–10), severe (11–14), or very severe (15–21). In the current sample, the HADS-D yields a Cronbach’s Alpha of 0.67 for the anxiety scale and 0.69 for the depression scale.

### Statistics

*Research question 1* For this analysis, the AROUSAL FACES TOTAL and the AROUSAL WORDS TOTAL scores were summed up to an AROUSAL TOTAL score. A multiple regression analysis (enter method) was performed with arousal elicited by multiple facial expressions and words (AROUSAL TOTAL) as dependent variable and age, gender, the six DERS scales, and the HADS-D depression and anxiety scales as independent variables. In the light of investigating shared predictors of both arousal types, the intensity ratings of facial expressions (INTENSITY FACES TOTAL) were omitted from this analysis, as they only apply to emotional faces and no equivalent was assessed for emotional words.

*Research question 2* A multiple regression analysis (enter method) was performed with arousal elicited by negative facial expressions (AROUSAL FACES NEGATIVE) as dependent variable and age, gender, intensity ratings of negative facial expressions (INTENSITY FACES NEGATIVE), arousal elicited by words (AROUSAL WORDS TOTAL), the six DERS scales, and the HADS-D depression and anxiety scales as independent variables.

*Research question 3* A multiple regression analysis (enter method) was performed with arousal elicited by neutral facial expressions (AROUSAL FACES NEUTRAL) as dependent variable and age, gender, intensity ratings of the neutral facial expressions (INTENSITY FACES NEUTRAL), arousal elicited by words (AROUSAL WORDS TOTAL), the six DERS scales, and the HADS-D depression and anxiety scales as independent variables.

*Research question 4* A multiple regression analysis (enter method) was performed with arousal elicited by positive facial expressions (AROUSAL FACES POSITIVE) as dependent variable and age, gender, intensity ratings of the positive facial expressions (INTENSITY FACES POSITIVE), arousal elicited by words (AROUSAL WORDS TOTAL), the six DERS scales, and the HADS-D depression and anxiety scales as independent variables.

*Research question 5* A multiple regression analysis (enter method) was performed with arousal elicited by negative words (AROUSAL WORDS NEGATIVE) as dependent variable and age, gender, arousal elicited by faces (AROUSAL FACES TOTAL), the six DERS scales, and the HADS-D depression and anxiety scales as independent variables.

*Research question 6* A multiple regression analysis (enter method) was performed with arousal elicited by neutral words (AROUSAL WORDS NEUTRAL) as dependent variable and age, gender, arousal elicited by faces (AROUSAL FACES TOTAL), the six DERS scales, and the HADS-D depression and anxiety scales as independent variables.

*Additional calculations regarding research questions 1*–*6* Whenever the dependent variable was significantly explained by a least one of the group variables gender or age (for age groups please see the results section where it applies), an ANCOVA with the predicted variable as dependent variable, the predicting group variable(s) as independent variable(s) and the other predictor(s) as covariate(s) (where it applies) were calculated.

*Research question 7* A two-step cluster analysis (log-likelihood distance, Schwarz–Bayes cluster criteria (BIC) was calculated so as to find distinctive subjective emotional arousal groups regarding this study. The variables age, gender, arousal elicited by multiple facial expressions (AROUSAL FACES TOTAL), arousal ratings elicited by emotional words (AROUSAL WORDS TOTAL), intensity ratings of the facial expressions (INTENSITY FACES TOTAL), the six DERS scales, and the HADS-D depression and anxiety scales were included in the cluster analysis. Subsequently, a discriminant analysis was performed so as to obtain classification accuracy rates for the distinctive groups found in the cluster analysis based on the previously mentioned variables. Furthermore, following the cluster analysis, multivariate group differences between cluster groups were calculated using a multivariate analysis of variance (MANOVA) with the previously determined cluster group affiliation as independent variable and age, arousal elicited by multiple facial expressions (AROUSAL FACES TOTAL), arousal ratings elicited by emotional words (AROUSAL WORDS TOTAL), intensity ratings of the facial expressions (INTENSITY FACES TOTAL), the six DERS scales, and the HADS-D depression and anxiety scales as dependent variables. Regarding those dependent variables for which significant differences between the cluster groups were shown, a Bonferroni post hoc analysis was performed. Differences regarding gender distribution across cluster groups were calculated with a Chi Square test.

Data handling and analyses were carried out using SPSS for Windows, Version 25.0. Correlations regarding dependent and independent variables are shown in Table 1. The *p* value for analyses regarding research questions 1–6 was set at 0.05; for research question 7 the Bonferroni–Holm method (Holm, 1979) for *p* value correction in the course of multiple testing was used (each corresponding corrected *p* value marked as p*). For the purpose of clarity all corrected *p* values will be presented, irrespective of the rejection criteria (first non-significant result).

**Table 1:**
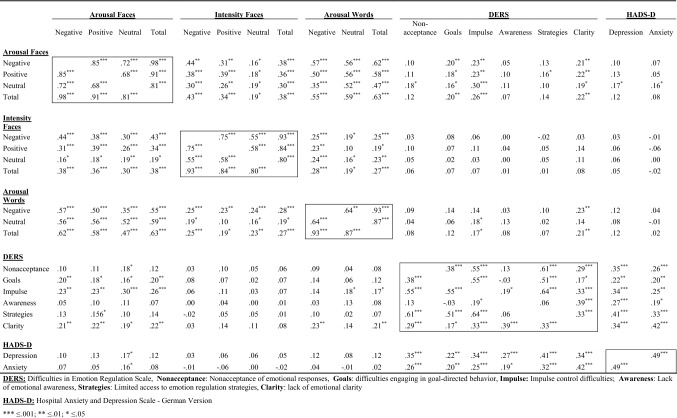
Pearson correlations between dependent and independent variables

## Results

### Research question 1: predictors of arousal elicited by multiple facial expressions and words

Results of the multiple regression analysis showed that one variable significantly predicted AROUSAL TOTAL, *F*(10,169) = 1.943, *p* = 0.043. 11%, *R*^*2*^ = 0.11, of the variation in the outcome was predicted by DERS factor clarity, *p* = 0.006*, b* = 42.058, *β* = 0.259. DERS factor IMPULSE, *p* = 0.069, did show a statistical trend. The variables gender, *p* = 0.403; age, *p* = 0.935; DERS factors: GOALS, *p* = 0.419, AWARENESS, *p* = 0.527, NON-ACCEPTANCE, *p* = 0.662, and STRATEGIES, *p* = 0.313; and the HADS-D scales depression, *p* = 0.349, and anxiety, *p* = 0.223, had no significant predictive effect.

### Research question 2: predictors of arousal elicited by negative facial expressions

Results of the multiple regression analysis showed that three variables significantly predicted AROUSAL FACES NEGATIVE, *F*(12,169) = 13.511, *p* ≤ 0.0001. 51%, *R*^*2*^ = 0.51, of the variation in the outcome was predicted by age, *p* = 0.045, *b* = − 0.543, *β* = − 0.120; AROUSAL WORDS TOTAL, *p* ≤ 0.0001, *b* = 0.148, *β* = 0.525, and INTENSITY FACES NEGATIVE, *p* ≤ 0.0001, *b* = 0.461, *β* = 0.289. The variables gender, *p* = 0.383; DERS factors IMPULSE, *p* = 0.286, GOALS, *p* = 0.561, AWARENESS, *p* = 0.580, NON-ACCEPTANCE, *p* = 0.622, STRATEGIES, *p* = 0.823, and CLARITY, *p* = 0.388; and the HADS-D scales depression *p* = 0.771, and anxiety, *p* = 0.683 had no significant predictive effect.

#### Additional calculations: research question 2

An additional ANCOVA with AROUSAL FACES NEGATIVE as dependent variable, age group (as suggested in literature by, e.g., Berk, 2014; Feldman, 2017; Steinberg, 2016; see the sample description) as independent variable and AROUSAL WORDS TOTAL as well as intensity faces negative as covariates showed no differences in arousal scores between age groups, *F*(4,168) = 1.735, *p* = 0.145, whereas this result was significantly adjusted by these covariates (AROUSAL WORDS TOTAL: *p* ≤ 0.0001 and INTENSITY FACES NEGATIVE: *p* ≤ 0.0001). The means for each group are as follows: emerging adulthood: *M* = 172.5 (SD = 67.1), early adulthood: *M* = 190 (SD = 78.5), first half of middle adulthood: *M* = 171.4 (SD = 76.6), second half of middle adulthood: *M* = 171.2 (SD = 88), late adulthood: *M* = 151.2 (SD = 78.2).

### Research question 3: predictors of arousal elicited by neutral facial expressions

Results of the multiple regression analysis showed that three variables significantly predicted AROUSAL FACES NEUTRAL, *F*(12,169) = 6.452, *p* ≤ 0.0001. 33%, *R*^*2*^ = 0.330 of the variation in the outcome was predicted by gender, *p* = 0.023*, b* = − 6.455, *β* = − 0.159, AROUSAL WORDS TOTAL, *p* ≤ 0.0001, *b* = 0.029, *β* = 0.387, and DERS factor IMPULSE, *p* = 0.003, *b* = 1.630, *β* = 0.288. The variables age, *p* = 0.357; INTENSITY FACES NEUTRAL, *p* = 0.174; DERS factors GOALS, *p* = 0.845, AWARENESS, *p* = 0.461, NON-ACCEPTANCE, *p* = 0.506, STRATEGIES, *p* = 0.058, and CLARITY, *p* = 0.697; and the HADS-D scales depression, *p* = 0.987, and anxiety, *p* = 0.085 had no significant predictive effect.

#### Additional calculations: research question 3

An additional ANCOVA with AROUSAL FACES NEUTRAL as dependent variable, gender as independent variable and AROUSAL WORDS TOTAL as well as DERS factor IMPULSE as covariates showed significant differences in arousal scores between gender groups, *F*(1,169) = 5.485, *p* = 0.020, whereas this result was significantly adjusted by these covariates (AROUSAL WORDS TOTAL: *p* ≤ 0.0001 and IMPULSE: *p* = 0.001). Males (*M* = 38.3, SD = 21.1) showed higher arousal scores that females (*M* = 31.7, SD = 19.1).

### Research question 4: predictors of arousal elicited by positive facial expressions

Results of the multiple regression analysis showed that three variables significantly predicted AROUSAL FACES POSITIVE, *F*(12,169) = 10.330, *p* ≤ 0.0001. 44%, *R*^*2*^ = 0.441, of the variation in the outcome was predicted by gender, *p* = 0.029, *b* = − 7.514, *β* = − 0.140, AROUSAL WORDS TOTAL, *p* ≤ 0.0001, *b* = 0.049, *β* = 0.488, and INTENSITY FACES POSITIVE, *p* ≤ 0.0001, *b* = 0.477, *β* = 0.289. The variables age, *p* = 0.954; DERS factors IMPULSE, *p* = 0.461, GOALS, *p* = 0.499, AWARENESS, *p* = 0.876, NON-ACCEPTANCE, *p* = 0.341, STRATEGIES, *p* = 0.350, and CLARITY, *p* = 0.533; and the HADS-D scales depression, *p* = 0.666, and anxiety, *p* = 0.673, had no significant predictive effect.

#### Additional calculations: research question 4

An additional ANCOVA with AROUSAL FACES POSITIVE as dependent variable, gender as independent variable and AROUSAL WORDS TOTAL as well as INTENSITY FACES POSITIVE as covariates showed no differences in arousal scores between gender groups, *F*(1,169) = 3.279, *p* = 0.072, whereas this result was significantly adjusted by these covariates (AROUSAL WORDS TOTAL: *p* ≤ 0.0001 and INTENSITY FACES POSITIVE: *p* ≤ 0.0001), males: *M* = 62.2 (SD = 24.6), females: *M* = 57.1 (SD = 29).

### Research question 5: predictors of arousal elicited by negative words

Results of the multiple regression analysis showed that two variables significantly predicted AROUSAL WORDS NEGATIVE, *F*(11,169) = 7.720, *p* ≤ 0.0001. 35%, *R*^*2*^ = 0.35 of the variation in the outcome was predicted by AROUSAL FACES TOTAL, *p* ≤ 0.0001, *b* = 0.794, *β* = 0.538, and DERS factor CLARITY, *p* = 0.038, *b* = 13.297, *β* = 0.170. The variables age, *p* = 0.129; gender, *p* = 0.081; DERS factors IMPULSE, *p* = 0.491, GOALS, *p* = 0.441, AWARENESS, *p* = 0.578, NON-ACCEPTANCE, *p* = 0.953, and STRATEGIES, *p* = 0.782; and the HADS-D scales depression, *p* = 0.344, and anxiety, *p* = 0.230 had no significant predictive effect.

### Research question 6: predictors of arousal elicited by neutral words

Results of the multiple regression analysis showed that two variables significantly predicted AROUSAL WORDS NEUTRAL, *F*(11,169) = 9.578, *p* ≤ 0.0001. 40%, *R*^*2*^ = 0.40 of the variation in the outcome was predicted by gender, *p* = 0.049; *b* = − 32.675, *β* = − 0.130, and AROUSAL FACES TOTAL, *p* ≤ 0.0001, *b* = 0.626, *β* = 0.571. The variables age, *p* = 0.695; DERS factors IMPULSE, *p* = 0.114, GOALS, *p* = 0.303, AWARENESS, *p* = 0.517, NON-ACCEPTANCE, *p* = 0.640, STRATEGIES, *p* = 0.457, and CLARITY, *p* = 0.827; and the HADS-D scales depression, *p* = 0.654, and anxiety, *p* = 0.239, had no significant predictive effect.

#### Additional calculations: research question 6

An additional ANCOVA with AROUSAL WORDS NEUTRAL as dependent variable, gender as independent variable and AROUSAL FACES TOTAL as covariate showed significant differences in arousal scores between gender groups, *F*(1,169) = 6.596, *p* = 0.011, whereas this result was significantly adjusted by the covariate (AROUSAL FACES TOTAL: *p* ≤ 0.0001). Males (*M* = 306.2, SD = 125) showed higher arousal scores that females (*M* = 259.2, SD = 121).

### Research question 7: arousal profiles

The cluster analysis with respect to the variables age, gender, AROUSAL FACES TOTAL; AROUSAL WORDS TOTAL; INTENSITY FACES TOTAL, the DERS factors IMPULSE, CLARITY, GOALS, AWARENESS, NON-ACCEPTANCE, STRATEGIES, and the HADS-D depression and anxiety scales yielded three distinctive groups of subjects (for details see Table 2). The following discriminant analysis showed significant differences between the three groups with respect to the first and the second discriminant function, 1st function: canonical correlation = 0.95, Wilks’ Lambda = 0.048, *χ*^*2*^ (26, *N* = 170) = 488.902, *p* ≤ 0.0001; 2nd function: canonical correlation = 0.74, Wilks’ Lambda = 0.446, *χ*^*2*^ (12, *N* = 170) = 129.92, *p* ≤ 0.0001. 79.1% of the first group, 98.2% of the second group and 100% of the third group were classified correctly, yielding an overall classification accuracy of 94.1% by the previously mentioned variables.

**Table 2:** Results from the two-step cluster analysis and the multivariate analysis with respect to the cluster groups

	Group I	Group II	Group III
	“Emotional difficulties disposition group”	“Low emotional difficulties group”	“Low emotional awareness group”
Sociodemographic variables			
*N*	43	57	70
*Females* (*frequency*)	*36*	*57*	*0*
*Males* (*frequency*)	*7*	*0*	*70*
Age (years)	43.3 (18.0)	46.1 (15.5)	43 (17.0)
Arousal ratings			
Arousal faces—total	284.6 (111.3)^t^	231 (116)^t^	275.7 (111.3)^t^
Arousal words—total	852.7 (271.2)^t^	717.2 (263.1)^t^	789.2 (259.4)^t^
Intensity ratings			
Intensity faces—total	431.8 (80.4)	428.2 (78.3)	427.4 (87.2)
DERS			
*Clarity*	*9.4* (*2.7*)	*7.1* (*1.6*)	*7.7* (*1.7*)
*Impulse*	*13.2* (*4.5*)	*7.7* (*1.7*)	*8.7* (*2.3*)
*Goals*	*15.9* (*4.7*)	*10.0* (*2.9*)	*11.2* (*3.5*)
*Awareness*	*14.5* (*4.5*)	*12.5* (*3.2*)	*14.8* (*3.7*)
*Nonacceptance*	*14.0* (*4.7*)	*9.6* (*2.6*)	*10.6* (*3.0*)
*Strategies*	*17.9* (*4.9*)	*11.3* (*1.9*)	*12.0* (*2.6*)
HADS-D			
*Depression*	*3.9* (*2.8*)	*1.4* (*1.3*)	*2.6* (*2.0*)
*Anxiety*	*6.0* (*2.1*)	*3.5* (*2.0*)	*4.1* (*2.4*)

The sociodemographic variables, arousal ratings for facial expressions and words, the intensity ratings for the face task, the difficulties in emotion regulation scale (DERS) scores as well as the Hospital Anxiety and Depression Scale—German Version (HADS-D) are presented. Scores are presented in means (standard deviations) and frequencies, respectively

Scores and ratings for which significant multivariate differences were found are given in italics

Variable range: arousal faces (75–675), arousal words—total (200–1800), intensity faces—total (75–675), clarity (5–25), impulse (6–30), goals (5–25), awareness (6–30), non-acceptance (6–30), strategies (8–40), depression and anxiety (0–7 no clinical signs, 8–10 borderline; participants with a score greater 10 were excluded from the analyses)

*DERS* Higher values mean greater difficulties in emotion regulation, *Impulse* impulse control difficulties, *Clarity* lack of emotional clarity, *Goals* difficulties engaging in goal-directed behavior, *Awareness* lack of emotional awareness, *Nonacceptance* non-acceptance of emotional responses, *Strategies* limited access to emotion regulation strategies

^t^Results for which statistical trends (MANOVA) were shown

The multivariate analysis of variance (MANOVA) yielded significant differences between cluster groups regarding DERS factors NON-ACCEPTANCE, *F*(11,158) = 22.374, *p* ≤ 0.0001(p* = 0.0041), GOALS, *F*(11,158) = 33.826, *p* ≤ 0.0001 (p* = 0.0045), IMPULSE, *F*(11,158) = 49.552, *p* ≤ 0.0001 (p* = 0.005), STRATEGIES, *F*(11,158) = 62.176, *p* ≤ 0.0001 (p* = 0.0055), CLARITY, *F*(11,158) = 18.108, *p* ≤ 0.0001 (p* = 0.0625), AWARENESS, *F*(11,158) = 6.152, *p* = 0.003 (p* = 0.01); and HADS-D scales depression, *F*(11,158) = 18.295, *p* ≤ 0.0001 (p* = 0. 0071), and anxiety, *F*(11,158) = 17.247, *p* = ≤ 0.0001 (p* = 0. 0083). Statistical trends were shown for AROUSAL FACES TOTAL, *F*(11,158) = 3.531, *p* = 0.031 (p* = 0.0125) and AROUSAL WORDS TOTAL, *F*(11,158) = 3.294, *p* = 0.040 (p* = 0.016). No significant differences were shown regarding age, *F*(11,158) = 0.597, *p* = 0.552 (p* = 0.025), and INTENSITY FACES TOTAL, *F*(11,158) = 0.041, *p* = 0.960 (p* = 0.05). The results of the post hoc analysis are shown in Table 3.

**Table 3:** Bonferroni post hoc analysis regarding variables for which significant group differences and statistical trends were shown in the MANOVA. *p* values are shown

Comparison group	Group I		Group II		Group III	
	“Emotional difficulties disposition group”		“Low emotional difficulties group”		“Low emotional awareness group”	
	Group II	Group III	Group I	Group III	Group I	Group II
Arousal ratings						
Arousal faces—total	.059^t^	.999	.059^t^	.083^t ^	.999	.083^t ^
Arousal words—total	.036^t^	.648	.036^t^	.382	.648	.382
DERS						
Clarity	≤ *.0001*	≤ *.0001*	≤ *.0001*	.391	≤ *.0001*	.391
Impulse	≤ *.0001*	≤ *.0001*	≤ *.0001*	.165	≤ *.0001*	.165
Goals	≤ *.0001*	≤ *.0001*	≤ *.0001*	.269	≤ *.0001*	.269
Awareness	*.034*	.999	*.034*	*.003*	.999	*.003*
Nonacceptance	≤ *.0001*	≤ *.0001*	≤ *.0001*	.334	≤ *.0001*	.334
Strategies	≤ *.0001*	≤ *.0001*	≤ *.0001*	.605	≤ *.0001*	.605
HADS-D						
Depression	≤ *.0001*	*.004*	≤ *.0001*	*.004*	*.004*	*.004*
Anxiety	≤ *.0001*	≤ *.0001*	≤ *.0001*	.376	≤ *.0001*	.376

*DERS* Difficulties in Emotion Regulation Scale, *Nonacceptance* non-acceptance of emotional responses, *Goals* difficulties engaging in goal-directed behavior, *Impulse* Impulse control difficulties, *Awareness* lack of emotional awareness, *Strategies* limited access to emotion regulation strategies, *Clarity* lack of emotional clarity, *HADS*-*D* Hospital Anxiety and Depression Scale—German Version

Statistical significant differences between the respective cluster groups regarding the respective variable are given in italics

^t^ Statistical trend

The Chi square test showed that gender was not equally distributed across cluster groups, *χ*^*2*^(2, *N* = 170) = 146.349, *p* ≤ 0.0001.

The identified cluster groups and group differences can be described as follows: whereas groups did only show a trend regarding differences in emotional arousal and no significant differences regarding age and intensity ratings, significant differences were shown regarding the DERS factors as well as subclinical depression and anxiety symptoms (see Tables 2 and 3). Group I (*emotional difficulties disposition group*), as the group with the by tendency highest arousal ratings, is characterized by relatively higher difficulties in emotion regulation in general, by relatively higher values in current subclinical depression and anxiety symptoms, and by a higher number of females in this group (84% of this cluster group). Group III (*low emotional awareness group*) shows by trend lower arousal ratings than group I and higher arousal ratings than group II. Furthermore, group III shows, by and large, lower emotion regulation difficulties as well as depression and anxiety symptoms than group I, whereas, similar to group I, they show higher problems in emotional awareness. Furthermore, group III shows, albeit not significantly, higher DERS and anxiety scores than group II, whereas it shows significantly higher depression scores than group II. Interestingly, this group consists exclusively of males. Group II (*low emotional difficulties group*) shows by tendency the lowest arousal ratings. It shows significantly lower emotion regulation difficulties than group I and significantly lower DERS emotional awareness scores than group III. Group II has the lowest values in depression symptoms as well as in anxiety symptoms (no statistical significant differences between groups II and III) and, again interestingly, consists exclusively of females.

## Discussion

Emotional arousal is an important aspect of everyday emotion processing and regulation (e.g., English & Carstensen, 2014; Phillips et al., 2003). To date, only a few studies addressed subjective emotional arousal in typically developing individuals, therefore this study aimed to offer new data with respect to this topic. Another aim was to investigate whether the results of the current study indicate some sort of dispositional baseline of subjective emotional arousal that transcends the processing of single stimulus types. Furthermore, this study investigated the predictive value of age, gender, intensity, emotion regulation difficulties, and depression and anxiety symptoms with respect to subjective emotional arousal in typically developing individuals across adulthood. The last aim was to look whether distinguishable subjective emotional arousal profiles can be found within the current sample and how these possible profiles could be interpreted in terms of a possible emotional personality style.

### Proposal of a dispositional baseline of emotional arousal

In the current study, strong associations between arousal elicited by facial expressions and arousal elicited by emotional words were found as both predicted each other. Although both constructs showed a very similar covariation across the cluster groups found in this study, it has to be noted that they did not share predictors, except for gender, which will be discussed further below. These results lead to the hypothesis that both arousal regarding facial expressions and emotional words are specialized parts of a greater emotional processing system (see e.g., the neurobiological model by Phillips et al., 2003) that comprises different modalities (see, e.g., amodal system of emotion perception in different communication channels by Borod et al., 2000). Given that this association was shown within a sample covering a great age span in adulthood (20–70 years), it can be hypothesized that this could be explained by some kind of stable, dispositional baseline of emotional arousal with respect to stimuli of different modalities like, for example, faces and words. In this context, Borod et al. (2000) showed positive correlations regarding emotion processing across facial, lexical and prosodic channels, and propose a general affective processor, at least with respect to the identification of emotional stimuli. They relate this general affective processor to previously published concepts describing some kind of emotional semantics in terms of conceptual knowledge of emotion (Adolphs et al. 1996; Bowers et al. 1993). The properties of such a modality-unspecific dispositional baseline—like, for example, stability—should be investigated in future studies. In this context, English and Carstensen (2014) showed that everyday emotional experience is significantly influenced by daytime. In the current study, it has to be noted that the time of day of the participation was not explicitly controlled. Nevertheless, the time of participation varied greatly throughout the day and the early evening which should have diminished such influence somehow.

### Possible effects of implicit emotion regulation regarding emotional arousal

In this study, it cannot be ruled out that implicit emotion regulation took place, especially given that the behavioral responses reflected the result of emotional processing under no time pressure. In this context, the authors of the current study propose two hypotheses regarding the strong association and similar covariation of both subjective emotional arousal scores found in this study. The first explanation could be that similar initial arousal levels at the stage of perceiving facial expressions and emotional words are regulated with the same strength regarding both stimuli types, leading to the individual’s similar levels of subjective emotional arousal. In short, individuals would show a baseline of initial similar levels of emotion arousal that is afterwards evenly regulated (early stage alignment hypothesis). The second explanation could be that at the stage of perceiving these emotional stimuli individuals show different levels of arousal which are afterwards adjusted by emotion regulation processes, leading to individual’s similar levels of subjective emotional arousal. This would allow the individuals to experience similar levels of emotional arousal under regular conditions, in short, a baseline of already regulated emotional arousal (late stage alignment hypothesis). Considering implicit and explicit components of emotion regulation in these theoretical considerations, both explanations would be theoretically supported by the neurobiological model of emotion processing by Phillips et al. (2003). They state a ventral neural system that is associated with autonomic responses and autonomic regulation as well as a dorsal system which is associated with rather cognitive aspects of emotion processing and the effortful regulation of affective states. As both systems show a close functional relationship (Phillips et al., 2003), it can be argued that adaptive processing (see, e.g., emotion-generative and -regulative cycles, “process model” by Gross and Thompson, 2007) theoretically enables both alignment procedures. In this context, strong associations between subjective and physiological arousal were shown (Alpers, Adolph, & Pauli, 2011; Balconi, Vanutelli, & Finocchiaro, 2014; Tan et al., 2016). Furthermore, judgements of arousal were previously shown to covary systematically with biological reflexes that are associated with the human defensive motive system (for an overview see Bradley et al., 2001a, b). Using behavioral, physiological, electrophysiological but also hemodynamic measures, future studies should investigate such proposed arousal baselines. Using event-related potentials and different paradigms, our research team currently investigates early (perceptual, automatic) and late (cognitively influenced) processing of emotional faces and words, represented by different ERP components, whereas this temporal high-resolution electrophysiological activity is compared to behavioral data of the participants. In the future, our research team will combine these measures with physiological and eye-tracking measures to investigate the relation between subjective and physiological arousal in more detail.

### The role of intensity

The subjective ratings of intensity of facial expressions were associated with subjective arousal for emotional faces (intensity and arousal ratings of positive faces; intensity and arousal ratings of negative faces) but not for neutral faces. The results regarding such associations between arousal and intensity regarding emotional stimuli are supported by previous behavioral (Deckert 2014; Goeleven et al. 2008) as well as by pharmacological studies (Beacher et al. 2011). Neutral faces typically elicit lowest arousal and intensity levels (see, e.g., Goeleven et al., 2008; Lundqvist et al., 1998), therefore the non-significant results regarding neutral expressions are most likely explained by floor effects. On the other hand, intensity did not significantly differentiate between the cluster groups found in this study. This indicates that the perception of intensity of facial expressions cannot be taken as a factor determining profiles of possible emotional personality styles.

### The role of emotion regulation difficulties

The results of the cluster analysis show a strong covariation between subjective emotional arousal scores and all emotion regulation difficulties scales. This indicates that higher values in stimulus-specific subjective arousal are associated with greater difficulties in the inhibition of impulsive behaviors, a decreased clarity regarding currently experienced emotions, difficulties in goal accomplishment, a higher tendency to have negative secondary emotional responses, a lower awareness regarding currently experienced emotions as well as a lower effective use of regulation strategies.

*The specific role of emotion clarity* In the regression analyses, the DERS factor “lack of emotion clarity”—the extent to which an individual is clear about the emotions he or she is currently experiencing—(Gratz & Roemer, 2004) significantly predicted the total arousal score (faces and words taken together) in the global regression but only arousal elicited by negative words in the type-specific regressions. In this context, emotional clarity seems to be an ability involved in meta-emotional knowledge, defined as a person’s declarative knowledge about emotions (Norman & Furnes, 2016) which is suggested to be a part of the umbrella term “meta-emotion”. Therefore, emotional clarity can be seen as an individual’s declarative knowledge about one’s own emotions. In this context, a previous study indicated that framing arousal words influence the rating of one’s emotional state (Bjalkebring & Johansson, 2015). Furthermore, an association between physiological indicators of arousal and emotional clarity (Williams et al., 2015) as well as between emotional clarity and arousal discrimination ability was shown (Nielsen, 2004). This result supports the definition of emotion regulation as involving “attention to and effortful regulation of arousal associated with affective states” (Phillips et al., 2003, pp. 509).

*The specific role of impulse control* In the regression analyses, the DERS factor “impulse control difficulties”—problems regarding the ability to inhibit inappropriate or impulsive behaviors—did show a statistical trend in the prediction of the total arousal score (faces and words taken together) in the global regression and significantly predicted exclusively arousal elicited by, interestingly, neutral faces. In the context of meta-emotion, the authors of the current study suggest that the impulse control factor of the DERS can be assigned to meta-emotional knowledge as well as meta-emotional experiences which can be seen as a subjective but not necessarily conscious component of meta-emotion (e.g., Norman & Furnes, 2016). Impulse control was already shown to be associated with indicators of physiological arousal (Williams et al., 2015) and with emotional modulation of response inhibition regarding emotional pictorial stimuli (Benvenuti et al., 2015). Considering the usual floor effect in arousal ratings regarding neutral expressions (e.g., Goeleven et al., 2008), it can therefore be hypothesized that those who have greater problems with inhibiting inappropriate behaviors show atypical perception, physiological reactions, and/or emotional response modulations with respect to neutral facial stimuli. In this context, it has to be noted that impulse control predicted arousal elicited by neutral facial expressions but not arousal elicited by (neutral) words. This result is supported by studies that show that compared to emotional words processing of facial expressions involves an enhanced sensory encoding for emotional content (Rellecke, Palazova, Sommer, & Schacht, 2011) and greater influence on early electrophysiological activity (Frühholz, Jellinghaus, & Herrmann, 2011), suggesting some kind of evolutionary preparedness for facial expressions (Rellecke, Palazova, Sommer, & Schacht, 2011, Vuilleumier & Pourtois, 2007). In this context, it can be assumed that facial expressions are more complex in a way that they carry more emotional information than written words without a context. At the same time, the different sources for emotional information in faces (e.g., gaze, eyebrows, brow, and mouth) potentially lead to a higher ambiguity in the perception of faces, especially in neutral faces as people differ, for example, in their default activation of facial muscles. This increased natural complexity as well as the supposed ambiguity in neutral faces would also support the previously hypothesized atypical emotional processing in individuals with greater impulse control difficulties. Future studies should investigate at which time point individuals with greater impulse control difficulties differ in their emotional responses (e.g., perception, reaction, regulation) using measures with high temporal resolutions such as event-related potentials. Furthermore, studies should investigate whether impulsivity predicts processing of (neutral) words when presented in different modalities or contexts.

The role of awareness will be discussed in the interpretation of the cluster groups below (see e.g., “Low emotional awareness group”).

### The role of age

In the present study, age did not distinguish between emotional arousal groups but, in conjunction with arousal elicited by words and subjective intensity ratings regarding negative faces, predicted arousal elicited by negative facial expressions (fear, anger, and sadness). This result is at least partially in line with previous literature with respect to age-related differences in emotional reactivity regarding arousing stimuli (e.g., Kessler & Staudinger, 2009), whereas especially negative stimuli tend to produce age differences (Gruehn & Scheibe, 2008). Nevertheless, an additional analysis investigating whether this age effect can also be seen in form of differences between explicit age groups, showed no significant differences between age groups. Furthermore, age did not predict arousal regarding words or other types of facial expressions. These results could potentially be explained as follows.

Negative faces seemingly elicit certain degrees of arousal for which age differences become apparent. In line with the supposed human preparedness for facial expressions (e.g., Rellecke et al., 2011; Vuilleumier & Pourtois, 2007), perceiving and interpreting negative faces seemingly and understandably holds a special role from an evolutionary point of view. Albeit not significant, results indicate that arousal regarding negative faces decreases with age and therefore rather follows age-specific changes in trait arousal (e.g., Kessler & Staudinger, 2009) and contradicts previous results on developmental changes in state emotional processing (e.g., Gruehn & Scheibe, 2008). Age in conjunction with another arousal parameter as well as perceived intensity of expressions was predictive of negative faces, while differences in arousal between explicit age groups were not shown. These results indicate complex interactions of influencing variables with respect to subjective arousal regarding negative faces.

Arousal regarding other stimulus types was not predicted by age. These results indicate that subjective arousal regarding relatively low arousing stimuli (for the comparison of the arousal-inducing potential of different types of stimuli see, e.g., the International affective picture system—IAPS, Lang, Bradley & Cuthbert, 1999) is not influenced by age. This further indicates that processing of relatively low arousing everyday stimuli is stable across the age span.

### The role of gender

In the present study, gender in conjunction with different arousal, intensity, and DERS scores was shown to predict arousal elicited by neutral and positive faces as well as by neutral words. Whereas for positive faces no significant gender group differences were shown in an additional analysis, it was shown that males yield significantly higher arousal scores for neutral stimuli than females. In this context, it was hypothesized that males rely less on subtle differences in facially expressed arousal when processing faces (Thayer & Johnsen, 2000) which could lead to over- or misinterpretations of neutral expressions. Furthermore, regarding facial emotion recognition, a moderate female superiority seems to exist (e.g., Donges, Kersting, & Suslow, 2012; Montagne et al., 2005; Andric-Petrovic et al., 2019; Thompson & Voyer, 2014), whereas this effect seemingly depends, inter alia, on the properties of the stimulus such as valence, specific emotion, gender of displayed face, or subtleness of emotion (e.g., Connolly, Lefevre, Young, & Lewis, 2019; Esposito et al., 2018; Hoffmann et al., 2010; Thompson & Voyer, 2014). The result regarding neutral words contradicts previous studies that showed no gender difference (Deckert, 2014). In this context, it is conceivable that the ambiguity of neutral words shows parallels to the ambiguity of neutral expressions (previously discussed in the “The specific role of impulse control” section), potentially leading to similar over- or misinterpretations.

In the present study, it was further shown that gender significantly distinguished between the cluster groups. It was shown that a number of females build the majority of the group that yields the highest arousal ratings (“emotional difficulties disposition group”), whereas the remaining females build the group with the lowest arousal ratings (“low arousal group”). Furthermore, the majority of males build the group with the intermediate arousal scores (“low emotional awareness group”), whereas the remaining males can be found in the group that yields the highest arousal ratings (“emotional difficulties disposition group”). Given this gender distribution across cluster groups, it can be hypothesized that the presence of different arousal types not only between gender groups but also within gender groups could at least partially explain incongruences regarding behavioral results and physiological as well as neuronal activation (e.g., Garnefski, Teerds, Kraaij, Legerstee, & van den Kommer, 2004; Kim, 2005; McRae et al., 2008). In this way, only a part of females would contribute to results such as that females are more reactive to unpleasant events and to show a broad disposition to respond with greater arousal to emotional stimuli, especially unpleasant ones (Bradley et al., 2001b; Gomez et al., 2013; Deng et al., 2016). Future studies on gender differences in emotion processing should take these different arousal types within gender groups into account.

### Emotional characteristics profiles

In the present study, three different cluster groups were identified. These groups did not differ significantly regarding perceived intensity of facial expressions or age. Furthermore, they did not differ significantly with respect to subjective emotional arousal which, strictly spoken, cannot lead to the interpretation of different emotional arousal profiles as noted in the aims section. As previously noted, it is conceivable that without a meaningful context, these everyday stimuli are possibly too low arousing for determining such profiles. Nevertheless, statistical trends can be seen with respect to subjective emotional arousal differences and will therefore be cautiously included in the interpretation.

Group one showed a high number of females (84% of this group) and had by trend the highest arousal scores as well as the significantly highest emotion regulation difficulties, and subclinical depression and anxiety scores. This group was called the “emotional difficulties disposition group” as the present combination of emotion-specific variables would in a higher severity presumably lead to serious emotional problems. Group three consisted exclusively of males and had by trend intermediate arousal scores, significantly lower scores in nearly all emotion regulation difficulties scores as well as lower subclinical depression and anxiety scores than group I. This group was called the “low emotional awareness group” as it showed by trend intermediate arousal scores but at the same time together with group I the lowest awareness regarding currently experienced emotions. Group two, on the other hand, comprised solely females and showed, overall, the lowest arousal, emotion regulation difficulties, depression, and anxiety scores (partly by trend, partly significant). It was therefore called the “low emotional difficulties group”.

Although similar levels of lack of emotional awareness could be found in the mainly female group one, group three showed the highest values in this emotion regulation difficulties score. In contrast to group one, group three, named the “low emotional awareness group”, consisted solely of males. This result is consistent with previous literature which showed higher scores on alexithymia scales in males, even in non-clinical samples (Levant et al., 2006, 2009).

These explorative results of the present study indicate that between as well as within gender groups, different emotional characteristics profiles can be found which highlight different aspects of emotion processing (e.g., disposition for emotional difficulties or low awareness for one’s own experienced emotions) which should definitely be investigated in future studies.

### Interpreting the results in the context of state–trait aspects of emotional processing

The superordinate aim of the study was to investigate immediate subjective arousal elicited by emotional stimuli and to look for influencing variables. It was shown that emotion regulation difficulties generally characterized specific emotional processing types which by trend include differences in subjective emotional arousal, whereas the aspects emotional clarity, impulse control, and emotional awareness seemingly have a specific influence on emotional arousal. In this line, it can be hypothesized that currently experienced “state” emotional arousal is influenced by personality aspects in terms of a trait “emotional personality style”. The existence of such stable relations between long-term emotional behaviors and current emotion processing are supported by recent studies (see, e.g., Kappes & Bermeitinger, 2016). With respect to such “emotional personality styles”, based on Labouvie-Vief’s “Model of affect optimization and affect complexity” (1998), Labouvie-Vief and Marquez (2004) showed four emotion processing groups which differed with respect to their ability to integrate positive and negative affect into flexible and differentiated structures as well as the ability to optimize their emotional experience based on this integration. Furthermore, linking emotional states with personality aspects, an imaging study showed associations between arousal and neuroticism as well as extraversion on a neuronal level (Kehoe et al., 2012). In the current study, the DERS scores lack of emotional clarity, impulse control, and emotional awareness can be interpreted as values of trait difficulties in emotion regulation as the instruction “Please indicate how often the following statements apply to you…” (Gole, Koechel, Schaefer, & Schienle, 2012) does not mention a specific time period for which this rating should be valid (e.g., the last 2 weeks). Given the results in this study, it can be argued that emotional clarity, impulse control, and emotional awareness potentially are trait aspects of emotion regulation which, together with gender, influence emotional arousal across a great part of adulthood. It is likely that further regulating or other mediating factors were not assessed in this study; therefore further studies regarding this topic are certainly required.

## Limitations

This study already included a sample with a great age span (20–70 years); nevertheless, future studies should additionally investigate younger as well as older participants so as to confirm current results and to add new insights. Although being a highly explorative study, some critical points regarding the task used in this study have to be noted.

First, the KDEF pictures showed faces of young adults, whereas the sample comprised young, middle and elder adults. Future studies should include pictures of different age groups.

Second, a number of facially expressed emotions were omitted. The authors of the current study decided to use a subset of emotional facial expressions that was previously validated for the population of Austria (Deckert, 2014) instead of stimuli that were not validated for this specific culture, as literature shows cultural differences in general as well as specifically regarding emotional face processing (e.g., Elfenbein & Ambady, 2002; Hills & Lewis, 2011). Future studies should also investigate further facial expressions like surprise, disgust, or contempt, whereas it was shown that these specific emotions are very frequently confused with other emotions (e.g., Calvo & Lundqvist, 2008; Ekman, 2003) and that, compared to the emotions chosen in this study, contempt is seen as an unbalanced facial expression as it is expressed by asymmetrical activation of facial muscles (Ekman, 2003).

Third, in this study, only negative and neutral words were investigated. Similar to the facial expressions used in this study, this subset of words was previously validated for the population of Austria (Deckert, 2014) and therefore chosen for this study so as to diminish possible cultural effects. For cultural differences in word processing see, for example, Redondo et al. (2007), Schmidt-Atzert and Park (1999), Soares et al. (2012), or Wierzbicka (2010).

Fourth, the results of the rating task used in this study likely represent a combined measure of emotional arousal and regulation. In this context, it has to be noted that a number of possible influential variables like, for example, the use of emotion regulation strategies (e.g., adaptive use, efficacy, etc.) were not controlled in this study. Future studies should address these shortcomings through the use of more elaborated tasks as well as the application of different behavioral, physiological, electrophysiological, and imaging techniques.

Fifth, the present study focused on the processing of facial expressions and words as they are omnipresent in everyday stimuli. Nevertheless, future studies should investigate other emotional stimuli like, for example, dynamic facial expressions, static or dynamic emotional body postures, and so on.

Sixth, this study tried to investigate the role of several variables that were previously associated with emotional arousal for which, so far, inconsistent results were shown. This was done in an explorative way, using a variety of tasks and analyses, and besides offering new data on this topic, this study itself produced ambiguous results which were discussed in the light of emotional arousal being a complex construct. Future studies should replicate and extend the methods used in this study to confirm its findings.

## Footnotes

1 The authors of the study want to thank the reviewers and the editor for the chance to add these scores to the analyses as well as for their valuable comments on it.
